# Longitudinal assessment of preoperative dexamethasone administration on cognitive function after cardiac surgery: a 4-year follow‐up of a randomized controlled trial

**DOI:** 10.1186/s12871-021-01348-z

**Published:** 2021-04-23

**Authors:** Sandro Glumac, Goran Kardum, Lidija Sodic, Cristijan Bulat, Ivan Covic, Mladen Carev, Nenad Karanovic

**Affiliations:** 1 Department of Anesthesiology and Intensive Care, University Hospital of Split, Spinciceva 1, 21000 Split, Croatia; 2 Department of Psychology, Faculty of Humanities and Social Sciences, University of Split, Split, Croatia; 3 Department of Neurology, University Hospital of Split, Split, Croatia; 4 Department of Cardiac Surgery, University Hospital of Split, Split, Croatia; 5 School of Medicine, University of Split, Split, Croatia; 6 Department of Anesthesiology and Intensive Medicine, School of Medicine, University of Split, Split, Croatia

**Keywords:** Cardiac Surgical Procedures, Postoperative Cognitive Complications, Cognitive Dysfunction, Neuropsychological Tests, Glucocorticoids, Dexamethasone

## Abstract

**Background:**

The pathogenesis of postoperative cognitive decline (POCD) is still poorly understood; however, the inflammatory response to surgical procedures seems likely to be involved. In addition, our recent randomized controlled trial showed that perioperative corticosteroid treatment may ameliorate early POCD after cardiac surgery. To assess the long-term effect of dexamethasone administration on cognitive function, we conducted a 4-year follow-up.

**Methods:**

The patients were randomized to receive a single intravenous bolus of 0.1 mg kg^− 1^ dexamethasone or placebo 10 h before elective cardiac surgery. The endpoint in both groups was POCD incidence on the 6th day and four years postoperatively.

**Results:**

Of the 161 patients analyzed previously, the current follow-up included 116 patients. Compared to the 62 patients in the placebo group, the 54 patients in the dexamethasone group showed a lower incidence of POCD on the 6th day (relative risk (RR), 0.510; 95 % confidence interval (CI), 0.241 to 1.079; *p* = 0.067, time interval also analyzed previously) and four years (RR, 0.459; 95 % CI, 0.192 to 1.100; *p* = 0.068) after cardiac surgery. The change in cognitive status between the two postoperative measurements was not significant (*p* = 0.010) among the patients in the dexamethasone group, in contrast to patients in the placebo group (*p* = 0.673).

**Conclusions:**

Although statistical significance was not reached in the current study, the prophylactic administration of dexamethasone seems to be useful to prevent POCD development following cardiac surgery. However, further large multicenter research is needed to confirm these directions.

**Trial registration:**

ClinicalTrials.gov identifier: NCT02767713 (10/05/2016).

**Supplementary Information:**

The online version contains supplementary material available at 10.1186/s12871-021-01348-z.

## Background

Postoperative cognitive decline (POCD) is a common complication following cardiac surgery [1]. Although the POCD rate drops over time, five years after cardiac surgery, the incidence remains up to 40 % [2, 3]. In addition to POCD deteriorating neurocognitive performance, it has also been related to increased mortality, impaired quality of life and premature retirement [4, 5].

POCD pathogenesis is still unclear [6, 7]. For decades, it was thought that POCD was a repercussion of introducing cardiopulmonary bypass [2]. However, beating heart surgery did not reduce POCD incidence [8]. Moreover, recent evidence suggests that the microembolic load during cardiac surgery is independent of cardiopulmonary bypass utilization [9, 10]. In addition, studies have shown that the intraoperative oxygen titration strategy and blood pressure target during cardiac surgery are not associated with the postoperative cognitive outcome [11–13]. Currently, possible mechanisms that may underlie the complex and multifactorial pathogenesis of POCD include surgical perfusion- and patient- and anesthesia-related risk factors [2, 5]. However, the inflammatory response initiated by the surgical procedure itself is recognized as an important factor in POCD development [5, 14, 15].

In that light, our previously published trial investigated the effect of administration of a single preoperative low dose of dexamethasone on cognitive outcomes in cardiac surgery patients. Compared to the placebo group, patients who received dexamethasone had a significantly reduced inflammatory response in terms of systemic inflammatory response syndrome and postoperative C-reactive protein levels and a significantly lower incidence of POCD [16]. The findings in the study by Valentin et al. support our results and show that the prophylactic application of 8 mg of dexamethasone improves cognitive outcomes up to six months after noncardiac surgery [17]. However, several other studies did not support our results [18–21], but they applied different corticosteroid treatment regimens and had different time points of cognitive evaluation. The major limitation of our trial was that cognitive functions were examined only during the early postoperative period [16].

Therefore, in the current follow-up of our previous trial, we sought to assess the longitudinal effects of preoperative dexamethasone administration on cognitive functions in patients who had undergone cardiac surgery. With reference to the findings from our previous trial, we hypothesized that patients who received dexamethasone would have better long-term cognitive outcomes than those who received placebo.

## Methods

### Study design

The current study represents a follow-up of a randomized, double-blind, placebo-controlled, parallel-arm trial [16] that was performed at the University Hospital of Split, Croatia, between March 2019 and January 2020. This paper adheres to the applicable Consolidated Standards of Reporting Trials guidelines and was conducted in accordance with the principles of the Declaration of Helsinki. Ethical approval for this study (Ethical Committee No. 2181-147-01/06/J.B.-16-2) was provided by the ethical committee of the University Hospital of Split (Chairperson Prof J. Bagatin) on 10 March 2015. All patients provided written informed consent. The original trial was registered at ClinicalTrials.gov (number: NCT02767713; date of registration: 10/05/2016).

### Study participants

The sample for the current research consisted of patients who were enrolled in our previous trial four years ago [16]. If the patients met any of the listed criteria during the follow-up period, they were excluded from the current study: cerebrovascular incident; neurodegenerative disease; mental illness; visual, hearing or motor impairment interfering with cognitive assessment; hospitalization due to heart failure; redo cardiac or carotid surgery; adrenal gland disease; corticosteroid treatment; and alcohol (> 20 g day^− 1^ or > 150 g week^− 1^) or controlled substance abuse.

### Study intervention and randomization

The full primary trial protocol has been published previously. Additionally, we have described patient randomization, perioperative management, acquisition procedures and statistical analysis and have previously reported primary and secondary study outcomes in detail [16]. Briefly, 161 patients aged between 41 and 84 years who were scheduled for elective coronary artery bypass graft surgery, heart valve surgery or combined surgery (coronary artery bypass graft surgery and valve surgery) were randomized (1:1) to receive a single intravenous bolus of 0.1 mg kg^− 1^ dexamethasone or the same volume of placebo (0.9 % NaCl) 10 h before surgery.

### Neuropsychological assessment

For the present study, the investigators employed the identical protocol and used the same validated battery of five neuropsychological tests, as fully described previously for neuropsychological assessment of the patients who participated in our trial four years ago [16]. In short, the evaluation was based on the following tests: Mini-Mental State Examination, Rey Auditory Verbal Learning Test (immediate and delayed recall), Wechsler Memory Scale and its three subscales (visual memory span, digit span forward, digit span backward), Symbol Digit Modalities Test and computerized PsychE (simple reaction time) test. Tests were administered to the patients two days before the surgical procedure, and then alternative forms of the tests were repeated on the 6th postoperative day. In the current study, each patient was invited via telephone call by nurse to the University Hospital of Split for late neuropsychological assessment precisely four years following cardiac surgery. The follow-up testing was conducted by the same trained neuropsychologist from the previous trial, who was blind to the treatment allocation of patients.

### Outcome measures

The primary endpoint of the current study was to assess the difference in POCD incidence four years after cardiac surgery between the dexamethasone and placebo groups. We also examined cognitive functions on the 6th postoperative day (time interval also analyzed in the previous study) in both groups and evaluated the relationship between the cognitive outcome at the two postoperative time points separately for the dexamethasone group and the placebo group. Again, we calculated the Jacobson and Truax reliable change index (RCI) for each patient. The RCI represents the sum of the Z-scores for the eight main variables. POCD in an individual patient was defined as an RCI equal to or less than − 1.96 or a Z-score equal to or less than − 1.96 on at least one test. In addition to the dichotomized cognitive test results at the different time points, we also directly compared continuous RCI values. A positive RCI value suggested improvement, whereas a negative value indicated deterioration in overall cognitive performance, as previously described [16].

### Statistical analysis

Data analysis was performed using IBM SPSS Statistics, version 24.0 (IBM Corp., Armonk, New York, USA). Categorical variables were expressed as numbers and percentages, and the chi-square test was used to determine the relationship between two categorical variables. Continuous variables are expressed as the means ± SD or medians and interquartile range, and independent-samples t test or Wilcoxon test was used to evaluate statistical significance. The independent-samples t test with Bonferroni adjusted *p* values was applied for comparisons of neuropsychological test results between the dexamethasone and placebo groups. Levene’s test to check the variance equality of the neuropsychological test results across samples was not significant. We calculated the relative risks (RRs) with 95 % confidence intervals (CIs) and tested the difference in POCD incidence at each postoperative time point between the dexamethasone and placebo groups using the chi-square test. Statistical significance was set at two-sided *p* < 0.05.

Given that the current study was the follow-up of a randomized controlled trial [16], no sample size calculation was performed.

## Results

### Study population

**Fig. 1:**
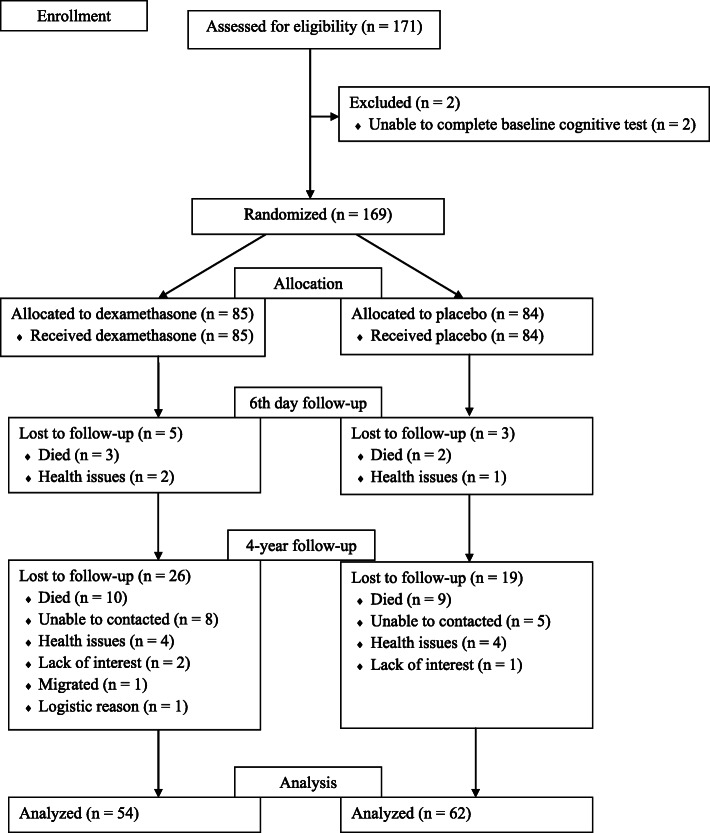
Patient enrollment flow chart. The flow of patients through this study investigated the long-term effect of dexamethasone on cognitive outcomes after cardiac surgery

Between March 2019 and January 2020, the current study recruited 116 patients (62 patients in the placebo group and 54 patients in the dexamethasone group) of the total 161 patients who were analyzed in our previous trial [16] between March 2015 and January 2016. The CONSORT flow diagram for the patients throughout the entire study is shown in Fig. 1. The reasons for loss to follow-up (in 45 patients) were death (19 patients, 42 %), our inability to contact the patient (13 patients, 29 %), health issues (8 patients, 18 %), lack of interest (3 patients, 7 %), and other reasons (2 patients, 4 %). The baseline demographic and clinical characteristics of the patients are presented in Table 1, whereas surgical and postoperative characteristics of the patients are presented in Supplemental Digital Content 1. There were no significant differences in any of the characteristics between the dexamethasone and placebo groups, apart from the length of the intensive care unit stay.

**Table 1:** Demographic and clinical characteristics

	Dexamethasone group *n* = 54	Placebo group *n* = 62	*p* value
Demographic characteristics			
Age (year)	67.4 (9.4)	67.1 (9.8)	0.900
Male sex	43 (79.6)	49 (79.0)	0.937
Body mass index (kg m^− 2^)	27.4 (3.3)	27.7 (3.4)	0.618
Elementary education	13 (24.0)	8 (12.9)	0.176
Secondary education	34 (63.0)	40 (64.5)	
Higher education	7 (13.0)	14 (22.6)	
Active smoking	18 (33.3)	21 (33.9)	0.946
Clinical characteristics			
General anesthesia	9 (16.7)	15 (24.2)	0.322
Hypertension	42 (77.8)	43 (69.4)	0.307
Insulin-dependent diabetes mellitus	3 (5.6)	4 (6.5)	0.294
Noninsulin-dependent diabetes mellitus	10 (18.5)	19 (30.6)	
Hyperlipidemia	40 (74.1)	47 (75.8)	0.830
Carotid artery disease	30 (55.5)	37 (59.6)	0.657
Peripheral vascular disease	6 (11.1)	6 (9.7)	0.800
Atrial fibrillation	10 (18.5)	9 (14.5)	0.561
Myocardial infarction	22 (40.7)	26 (41.9)	0.896
Left ventricular ejection fraction (%)	60.8 (13.0)	60.6 (10.8)	0.924

Levels of education according to the Ministry of Science, Education and Sports of the Republic of Croatia range from elementary to secondary and higher education. General anesthesia refers to its administration within the past five years before cardiac surgery

Data are presented as numbers (%) or means ± SD as appropriate

### Cognitive outcomes

In the current analysis, on the 6th postoperative day (time interval also analyzed in the previous study), eight of the 54 (14.8 %) patients in the dexamethasone group and 18 of the 62 (29.0 %) patients in the placebo group fulfilled the diagnostic criteria for POCD. At the 4-year follow-up, six of the 54 (11.1 %) patients in the dexamethasone group and 15 of the 62 (24.2 %) patients in the placebo group fulfilled the diagnostic criteria for POCD. The difference in the POCD incidence between groups at the 6th postoperative day was RR, 0.510; 95 % CI, 0.241 to 1.079; *p* = 0.067, whereas at the 4-year follow-up, it was RR, 0.459; 95 % CI, 0.192 to 1.100; *p* = 0.068 (Table 2).

**Table 2:** Neuropsychological test results, reliable change index and POCD incidence

Test	Baseline		*p* value	POD 6		*p* value	4-year follow-up		*p* value
	Dexa	Placebo		Dexa	Placebo		Dexa	Placebo	
Mini-Mental State Examination	28.1 ± 1.3	28.3 ± 1.1	0.182	28.0 ± 1.3	27.5 ± 1.4	0.046	27.2 ± 2.5	27.2 ± 2.3	0.945
RAVLT -Immediate recall	41.2 ± 8.9	42.2 ± 8.8	0.547	36.5 ± 8.9	37.2 ± 11.3	0.725	33.6 ± 9.2	33.8 ± 10.1	0.906
RAVLT - Delayed recall	7.6 ± 3.2	7.6 ± 3.1	0.922	7.2 ± 11.1	5.4 ± 3.2	0.249	6.5 ± 2.6	5.9 ± 3.0	0.225
Visual memory span	9.8 ± 3.6	9.7 ± 3.2	0.855	9.4 ± 3.6	9.3 ± 3.8	0.787	9.9 ± 3.1	10.3 ± 3.1	0.519
Digit span forward	7.0 ± 1.2	7.0 ± 1.2	0.894	7.0 ± 1.2	6.8 ± 1.2	0.580	6.7 ± 1.4	6.7 ± 1.4	0.820
Digit span backward	4.9 ± 1.5	5.1 ± 1.2	0.415	4.9 ± 1.7	4.8 ± 1.5	0.776	4.4 ± 1.7	4.4 ± 1.6	0.941
Symbol Digit Modalities Test	31.8 ± 11.8	33.2 ± 10.2	0.505	29.6 ± 11.4	28.5 ± 11.2	0.616	32.4 ± 11.8	32.2 ± 12.1	0.947
Simple reaction time	1300.0 ± 365.1	1278.8 ± 460.5	0.784	1347.1 ± 450.1	1444.3 ± 540.7	0.293	1576.9 ± 531.0	1840.8 ± 832.8	0.042
Reliable change index				0.564 ± 1.5	-0.075 ± 1.0	0.007	0.296 ± 0.8	-0.017 ± 1.0	0.075
POCD incidence				8 (14.8)	18 (29.0)	0.067	6 (11.1)	15 (24.2)	0.068

Higher scores indicate better test performance except for simple reaction time, for which shorter time indicates better performance

The reliable change index is the sum of the Z-scores for the different tests. A positive value suggests an improvement, whereas a negative value suggests a decline in overall cognitive performance

POD 6, 6th postoperative day; Dexa, dexamethasone group; RAVLT, Rey Auditory Verbal Learning Test; POCD, postoperative cognitive decline

Data are presented as numbers (%) or means ± SD as appropriate

The longitudinal analysis of POCD incidence was conducted at two postoperative time points (i.e., on the 6th postoperative day and four years after cardiac surgery) separately for the dexamethasone group and for the placebo group. The purpose was to investigate how many patients with late POCD occurrence did not have an early POCD occurrence in each group. The analysis revealed that at the 4-year follow-up, 13 patients who did not have early POCD developed late POCD. Significantly, ten of those 13 patients were in the placebo group. Therefore, this study demonstrated that the constancy of POCD incidence over time in the patients in the dexamethasone group was statistically significant (*p* = 0.010), in contrast to the patients in the placebo group (*p* = 0.673).

The patient scores on the different tests and the RCI values are presented in Table 2. Analysis of the neuropsychological test battery results showed significant differences only in the Mini-Mental State Examination score on the 6th postoperative day (*p* = 0.046) and simple reaction time at four years (*p* = 0.042) after cardiac surgery between the dexamethasone and placebo groups. However, the difference in RCI values between groups on the 6th postoperative day was significant (*p* = 0.007), whereas at the 4-year follow-up, the difference was *p* = 0.075.

## Discussion

The current follow-up study including 116 patients supported and expanded the results of our previous randomized controlled trial on the beneficial effects of preoperative dexamethasone administration on postoperative cognitive function in cardiac surgery patients. To our knowledge, this is the first study to evaluate the long-term effect of perioperative corticosteroid treatment on cognitive outcomes after cardiac surgery.

Our previous trial showed that patients who preoperatively received dexamethasone had a significantly reduced inflammatory response induced by cardiac surgery and significantly decreased the risk of early POCD in comparison to those who received placebo (11.3 versus 25.9 %) [16]. However, whether there is a prolonged beneficial effect of dexamethasone on cognitive status remains unresolved. Therefore, in this study, we extended the scope of our previous trial by using a longer period of follow-up. The time interval for cognitive assessment was set at four years after cardiac surgery for two main reasons. First, if we assessed patients earlier (e.g., at one year after cardiac surgery), it would probably not properly reflect the long-term effect of cardiac surgery and dexamethasone administration on cognitive functions. Second, if the cognitive evaluation occurred more than five years after surgery, a substantial number of different factors, such as natural aging or the development of dementia, may interfere with the study results, and therefore, in our opinion, that type of investigation can be reserved only for large multicenter research. Additionally, it should be advised that in the case of multiple testing, the learning effect among patients is considerable [2, 5]. According to our current results, POCD incidence was twofold lower in the dexamethasone group on the 6th postoperative day as well as at the 4-year follow-up than in the placebo group. Importantly, the similarity of the obtained confidence intervals on the 6th postoperative day and four years after cardiac surgery suggests the reliability of the study results. Furthermore, significantly fewer patients in the dexamethasone group than in the placebo group had a change in their cognitive status over time. In particular, only three patients in the dexamethasone group developed late POCD without early POCD. Thus, evidence suggests that preoperative dexamethasone treatment results in a low rate of POCD incidence at the 4-year follow-up by attenuating the inflammatory response, decreasing the risk of early POCD and subsequently maintaining a stable patient cognitive status for a prolonged period. However, the interpretation of the results should be approached with caution considering that the sample size was relatively small, particularly when the longitudinal design of the current study is taken into account. Therefore, only large multicenter studies can provide a final judgment about the possible long-term beneficial effect of preoperative dexamethasone administration on cognitive impairment following cardiac surgery.

Analysis of the neuropsychological test battery results showed significant differences in the Mini-Mental State Examination score on the 6th postoperative day and simple reaction time at four years after cardiac surgery between the dexamethasone and placebo groups. Thus, the analysis revealed that patients in the dexamethasone group better organized and stored verbal content in memory and then more efficiently recalled the retained verbal material from memory. Additionally, patients in the dexamethasone group were quicker in perception and response execution, i.e., in the domain of psychomotor speed. Relander et al. showed that the most consistently impaired cognitive domain during six years of follow-up after cardiac surgery was executive functioning, probably because of the complex and widespread neurocognitive network necessary for daily functioning [22]. Our results suggest that the prophylactic administration of dexamethasone is particularly effective at preserving this area of cognitive performance following cardiac surgery.

Several recent studies have reported the uselessness of perioperative corticosteroid administration on cognitive functions following cardiac surgery. Notably, the cognitive assessments in previous studies were conducted only up to one year following surgery. The discrepancy in the results probably lies in the different corticosteroid treatment regimens among studies [18–21]. As mentioned in our previous paper, we believe that the selection of dexamethasone as a potent synthetic glucocorticoid with a long duration of action, the administration of a low dose to avoid toxic effects on neural structures, and well-timed administration to provide anti-inflammatory effects throughout the early perioperative period are vital to prevent cognitive impairment after cardiac surgery [16]. Moreover, a recent study also reported that corticosteroids had a protective effect on cognitive functions after noncardiac surgery, based on the same type and dose of corticosteroids as in the present study [17].

The current study has several limitations that have already been discussed in our previous paper [16]. In addition, although we did our best to identify and exclude patients with such difficulties, the effects of natural aging, the progression or new silent incidence of cerebrovascular disease, or the development of dementia may have interfered with neuropsychological test results at the 4-year follow-up [2, 23]. Furthermore, the attrition rate of patients in this study was 28 %, which is comparable with the rates in similar studies [3, 12]. The exclusion of dropouts in our analysis could be perceived as a bias, but the reasons for withdrawing from longitudinal studies are usually very different, including death, inability to contact participants, health issues, lack of interest, migrations and logistics. Patient death was the most common reason for losing data in this study, and 14 of the 19 deaths were caused by different types of cancer that patients developed during the study period. Because the studies that long-term evaluate cognitive functions after surgery face these difficulties, we believe that there is no scientific grounding for the inclusion of such patients in the current analysis as POCD cases. Many colleagues share this opinion, given that Ottens et al. calculated missing data in their longitudinal study as POCD cases at only 1 month of follow-up, when the reasons for dropping out were far fewer than at 1 year of follow-up [18], whereas Larsen et al. completely ignored dropouts in their 3-year follow-up analysis [12]. Next, we did not perform a POCD subanalysis according to different patient age groups because of insufficient sample size for that additional analysis. Also, some might argue that the choice of anesthetic technique may have a crucial impact on POCD development, but considering recent evidence, it is premature to conclude that any mode of anesthetic regimen is superior; therefore, future studies are required to determine whether a particular anesthetic approach has a more favorable risk profile for cognitive function [5, 24, 25]. Finally, we would like to emphasize that even though the sample size in this study was determined by a single hospital setting and with respect to obstacles in defining POCD among researchers [5, 26, 27], we believe that these preliminary results were fairly reported and broadly disseminated.

## Conclusions

It seems that the results of the current study further support the role of the inflammatory response to cardiac surgery as an important figure in the complex and mutually interdependent mechanisms that underlie POCD development during the perioperative period. However, the study conclusions are limited by the relatively small sample size and by not reaching statistical significance. Nevertheless, we hope that our findings will encourage worldwide POCD investigators to pool their strengths for conducting large multicenter research that will thoroughly examine a possible long-term benefit of preoperative dexamethasone administration on such significant postoperative outcomes as cognitive decline.

## Supplementary information

Additional file 1 **TableS1.** Surgical and postoperative characteristics

## Data Availability

The datasets used and analyzed during the current study are available from the corresponding author on reasonable request.

## Abbreviations

CI: Confidence interval
POCD: Postoperative cognitive decline
RCI: Reliable change index
RR: Relative risk
SD: Standard deviation
