# Supplementary material for: Longitudinal assessment of preoperative dexamethasone administration on cognitive function after cardiac surgery: a 4-year follow‐up of a randomized controlled trial
Source: BMC Anesthesiol. 2021 Apr 23;21:129. doi: 10.1186/s12871-021-01348-z (PMC8063389; doi:10.1186/s12871-021-01348-z)
Supplement: Supplementary file 1 — TableS1. Surgical and postoperative characteristics [file 12871_2021_1348_MOESM1_ESM.doc]

**Longitudinal assessment of preoperative dexamethasone administration on cognitive function after cardiac surgery: A 4-year follow-up of a randomized controlled trial**

Sandro Glumac, Goran Kardum, Lidija Sodic, Cristijan Bulat, Ivan Covic, Mladen Carev and Nenad Karanovic

**Supplemental Digital Content 1**

| **Table** Surgical and postoperative characteristics | | | |
| --- | --- | --- | --- |
|  | Dexamethasone group *n* = 54 | Placebo group *n* = 62 | *p* value |
| Surgical characteristics |  |  |  |
| CABG | 37 (68.5) | 41 (66.1) | 0.609 |
| Heart valve surgery | 13 (24.1) | 13 (21.0) |
| CABG and valve surgery | 4 (7.4) | 8 (12.9) |
| Surgery with CPB | 29 (53.7) | 29 (46.8) | 0.457 |
| CPB duration (min) | 82.0 (25.0) | 94.0 (58.0) | 0.053 |
| Cross-clamp duration (min) | 54.0 (21.8) | 58.0 (30.0) | 0.147 |
| Lowest bispectral index | 30.3 (7.4) | 29.7 (7.3) | 0.657 |
| Lowest temperature (ºC) | 34.4 (1.8) | 34.2 (2.4) | 0.583 |
| Lowest MAP (mmHg) | 55.0 (9.1) | 56.9 (9.1) | 0.257 |
| Lowest hematocrit (%) | 27.8 (6.4) | 28.3 (6.2) | 0.662 |
| Insulin administered | 4 (7.4) | 2 (3.2) | 0.552 |
| Vasopressor administered | 28 (51.9) | 40 (64.5) | 0.167 |
| Inotropic agent administered | 26 (48.1) | 29 (46.8) | 0.882 |
| Blood transfusion (ml) | 485.0 (775.5) | 285.0 (1180.5) | 0.669 |
| SVRi (dyne s cm^-5^ m^-2^) | 1401.0 (434.6) | 1515.3 (514.3) | 0.199 |
| Surgery duration (min) | 210.5 (56.1) | 227.8 (62.2) | 0.117 |
| Postoperative characteristics |  |  |  |
| Drainage in the first 12 h (ml) | 300.0 (272.5) | 390.0 (326.3) | 0.104 |
| Duration of m.v. in the ICU (h) | 17.5 (8.7) | 18.7 (8.8) | 0.440 |
| Time to extubation (h) | 18.5 (9.4) | 19.9 (9.1) | 0.409 |
| Length of ICU stay (h) | 45.3 (21.4) | 47.3 (36.1) | 0.036 |
| Length of hospital stay (day) | 11.0 (3.0) | 11.1 (2.9) | 0.891 |

CABG, coronary artery bypass graft surgery; CPB, cardiopulmonary bypass; MAP, mean arterial pressure; SVRi, systemic vascular resistance index (value is the index value at the end of the surgical procedure); m.v., mechanical ventilation; ICU, intensive care unit.

Data are presented as numbers (%), means ± SD, or medians (IQR) as appropriate.
